# Regulators of Human White Adipose Browning: Evidence for Sympathetic Control and Sexual Dimorphic Responses to Sprint Interval Training

**DOI:** 10.1371/journal.pone.0090696

**Published:** 2014-03-06

**Authors:** Rebecca L. Scalzo, Garrett L. Peltonen, Gregory R. Giordano, Scott E. Binns, Anna L. Klochak, Hunter L. R. Paris, Melani M. Schweder, Steve E. Szallar, Lacey M. Wood, Dennis G. Larson, Gary J. Luckasen, Matthew S. Hickey, Christopher Bell

**Affiliations:** 1 Department of Health and Exercise Science, Colorado State University, Fort Collins, Colorado, United States of America; 2 Heart Center of the Rockies, University of Colorado Health, Fort Collins, Colorado, United States of America; University of Warwick – Medical School, United Kingdom

## Abstract

The conversion of white adipose to the highly thermogenic beige adipose tissue has been proposed as a potential strategy to counter the unfavorable consequences of obesity. Three regulators of this conversion have recently emerged but information regarding their control is limited, and contradictory. We present two studies examining the control of these regulators. Study 1: In 10 young men, the plasma concentrations of irisin and fibroblast growth factor 21 (FGF21) were determined prior to and during activation of the sympathetic nervous system via hypoxic gas breathing (FIO_2_ = 0.11). The measurements were performed twice, once with and once without prior/concurrent sympathetic inhibition via transdermal clonidine administration. FGF21 was unaffected by basal sympathetic inhibition (338±113 *vs*. 295±80 pg/mL; *P* = 0.43; mean±SE), but was increased during hypoxia mediated sympathetic activation (368±135); this response was abrogated (*P* = 0.035) with clonidine (269±93). Irisin was unaffected by sympathetic inhibition and/or hypoxia (*P*>0.21). Study 2: The plasma concentration of irisin and FGF21, and the skeletal muscle protein content of fibronectin type III domain containing 5 (FNDC5) was determined in 19 young adults prior to and following three weeks of sprint interval training (SIT). SIT decreased FGF21 (338±78 *vs*. 251±36; *P* = 0.046) but did not affect FNDC5 (*P* = 0.79). Irisin was decreased in males (127±18 *vs*. 90±23 ng/mL; *P* = 0.045) and increased in females (139±14 *vs*. 170±18). Collectively, these data suggest a potential regulatory role of acute sympathetic activation pertaining to the browning of white adipose; further, there appears to be a sexual dimorphic response of irisin to SIT.

## Introduction

The conversion of white adipose tissue to the highly thermogenic beige adipose tissue has been identified as a potential strategy to counter the unfavorable consequences of obesity in adult humans [1]–[3]. Two regulators of this conversion have recently been described but information regarding their control is limited, and somewhat contradictory. Fibroblast growth factor 21 (FGF21) is released from the liver, white adipose tissue, and brown adipose tissue [4], [5], and is favorably associated with increased insulin sensitivity and protection from weight gain [6]–[9]. Similarly, irisin, the circulating product of the membrane bound protein, fibronectin type III domain containing 5 (FNDC5) found predominantly in skeletal muscle but also in white adipose tissue [4], [5], ; is also positively related to insulin sensitivity and weight loss [10]. Both FGF21and irisin/FNDC5 have been mechanistically linked to the conversion of white to beige adipose tissue [5], [10].

In light of the potential clinical significance of FGF21 and irisin/FNDC5 for the treatment of obesity, an understanding of their regulation is of obvious importance. In this regard, we have considered two potential regulators/perturbations that are not necessarily mutually exclusive: the sympathetic nervous system and sprint interval exercise training. Stimulation of sympathetic activity via cold exposure is a well-known activator of beige fat in humans [3], [12]–[14]. There is some evidence from experimental animals suggesting that the sympathetic nervous system may also have a direct regulatory role pertaining to FGF21 [4], [5], and indirectly to irisin/FNDC5 via stimulation of peroxisome proliferator-activated receptor gamma co-activator 1-alpha (PGC-1α; [15]). Further, exercise, perhaps via intermittent increased sympathetic activation, may also stimulate FGF21 and/or irisin/FNDC5, although the evidence is somewhat contradictory depending on species and type of exercise [10], [16]–[18].

In the current manuscript we present two studies. Study 1 is a retrospective analysis of plasma collected during a previously published study [19]; we address the new hypothesis that the sympathetic nervous system is an important physiological regulator of FGF21 and irisin in adult males. In Study 2 we performed a prospective investigation in which we examined the hypothesis that short-term sprint interval training would increase skeletal muscle FNDC5 protein content, and increase circulating concentrations of irisin and FGF21 in adult males and females.

## Methods

### Research Participants – Studies 1 and 2

The experimental protocols conformed to the standards set by the Declaration of Helsinki of 1975, as revised in 1983, and were approved by the Institutional Review Board at Colorado State University. The nature, purpose and risks of each study were explained to research participants before written informed consent was obtained. Select physiological characteristics of research participants from each study are presented in Table 1. One participant from Study 1 participated in Study 2.

**Table 1:** Selected baseline physical characteristics of research participants.

	Study 1	Study 2
Sex (M/F)	10/0	7/12
Age (years)	23±1	24±1
Body mass index (kg/m^2^)	24.2±0.8	26.8±1.1
% Body Fat	18.5±1.2	33.7±1.5
VO_2max_ (ml/kg/min)	46.8±2.5	35.4±1.5

Data are mean ± SE. VO_2max_: Maximal oxygen consumption.

### Procedures Common to Both Studies - Screening

Body composition was assessed using dual-energy x-ray absorptiometry (DXA-IQ; Lunar Radiation Corp., Madison, WI, software v. 4.1). Maximal oxygen uptake (VO_2max_) was determined with a metabolic cart (Parvo Medics, Sandy, UT) during incremental cycle ergometer or treadmill exercise to volitional fatigue, as previously described [20].

### Study 1 – Influence of the sympathetic nervous system on circulating FGF21 and irisin

Study 1 represents a retrospective analysis of plasma collected during a previously published study [19], the focus of which was insulin sensitivity, hypoxia and the sympathetic nervous system. Following screening, research participants (young, healthy adult males) reported to the laboratory on two separate occasions, following an overnight fast and 48-hour abstention from vigorous physical activity. On arrival participants were instrumented for measurement of heart rate, blood pressure, and oxyhemoglobin saturation (pulse oximeter; Cardiocap 5, GE Datex-Ohmeda, Madison, WI, USA) and a catheter was inserted into a dorsal hand vein for subsequent blood sampling. Following a brief period of quiet rest in a semi-recumbent position, blood (15 ml) was sampled and heart rate, blood pressure, and oxyhemoglobin saturation were recorded. To activate the sympathetic nervous system, research participants began breathing a hypoxic gas mixture (FIO_2_  =  0.11, balance Nitrogen). After 15 minutes, blood was sampled again and heart rate, blood pressure, and oxyhemoglobin saturation were re-recorded.

To determine the influence of basal sympathetic activity on circulating FGF21 and irisin, 48-hours prior to one of visits (random order) transdermal clonidine administration (Catapres-TTS; 0.2 mg/day) was initiated and continued for the duration of that visit. Clonidine is a blood pressure medication; the mechanism of action is via pre-junctional stimulation of alpha-2-adrenergic receptors. We, and others, have previously demonstrated that short-term clonidine use (2-to-7 days) results in centrally mediated peripheral sympathetic inhibition, as reflected by decreased plasma norepinephrine concentration and release, and attenuated skeletal muscle sympathetic nerve activity [21], [22].

Blood was divided equally into two, pre-chilled tubes, containing either K3-ethylenediaminetetraacetic acid (EDTA) or ethylene glycol tetraacetic acid/glutathione. Within 60 minutes of collection blood was centrifuged to isolate plasma that was subsequently stored at −80°C until analysis. Plasma concentrations of FGF21 (BioVender, Ashville, NC, USA), irisin (Phoenix Pharmaceutical, Burlingame, CA, USA), and catecholamines (Rocky Mountain Diagnostics, Colorado Springs, CO, USA) were measured using enzyme-linked immunosorbent assays (ELISA).

### Study 2 - Influence of short-term sprint interval training on skeletal muscle FNDC5 protein content and circulating FGF21 and irisin

Following screening research participants (young healthy adult males and females) completed nine sessions of sprint interval training over three weeks. Each session consisted of between four and eight, 30-second bouts of “all-out” maximal efforts separated by four minutes of recovery; to facilitate recuperation, each session was separated by one to two days.

To document the effectiveness of the sprint interval training, participants completed a time-to-exhaustion trial prior to and following the nine sessions. These trials consisted of a 15-minute low-intensity warm-up that transitioned into exercise at 80% of pre-training VO_2max_ (confirmed with indirect calorimetry at 5-minutes) until exhaustion. Exhaustion was defined as an inability to maintain the required external power output and/or volitional exercise cessation. Prior to the pre-training time to exhaustion trial, all research participants completed an identical trial for purposes of habituation. During all of these trials, time cues were obscured from the participants' field of vision.

Skeletal muscle and venous blood samples were collected prior to and following sprint interval training, after a 12-hour fast and 48-hour abstention from exercise. Skeletal muscle biopsies of the vastus lateralis were obtained under local anesthesia (1% lidocaine) using Bergstrom needles. Samples were flash frozen and stored at −80°C for further analysis. Skeletal muscle homogenates were obtained from powderized tissue samples lysed with phosphatase and protease inhibitors (HALT). All samples were standardized by total protein and analyzed by SDS-PAGE. Tissue FNDC5 (Abcam (ab93373), Cambridge, MA, USA) was determined by western blotting. Plasma concentrations of FGF21 and irisin were analyzed following the procedures described in Study 1. In light of previous reports of associations between insulin sensitivity and FGF21 and irisin, pre-training circulating concentrations of glucose and insulin were determined and used to calculate a value for homeostatic model assessment of insulin resistance (HOMA-IR: fasting glucose (mmol/L) × fasting insulin (mU/L)/22.5). Glucose concentration was analyzed immediately (from ∼1 ml whole blood) using an automated device (2300 STAT Plus Glucose Lactate Analyzer, YSI Inc., Yellow Springs, Ohio, USA) and insulin concentration via ELISA (ALPCO Diagnostics, Salem, NH, USA). In addition, our laboratory has an interest in the adipokine, pigment epithelium derived factor (PEDF), a physiological determinant of insulin sensitivity, oxidative stress and inflammation [19]–[21], [23]. Plasma PEDF concentration was also determined via ELISA (Millipore Corporation, Billerica, MA, USA).

### Statistical Analysis

These were controlled, repeated measures studies. Accordingly, main effects of, and interactions between, hypoxia and sympathetic inhibition, and sprint interval training and sex were examined via two-way repeated measures analysis of variance (ANOVA). Multiple comparisons of factor means were performed using the Newman-Keuls test. Paired sample t-tests were used to assess delta values for FGF21 and irisin. Pearson correlations were used to investigate relationships between the dependent variables and potential modifiers. The level of statistical significance was set at *P*<0.05. Data are expressed as mean ± SE.

## Results

### Study 1 – Influence of the sympathetic nervous system on circulating FGF21 and irisin

**Figure 1:**
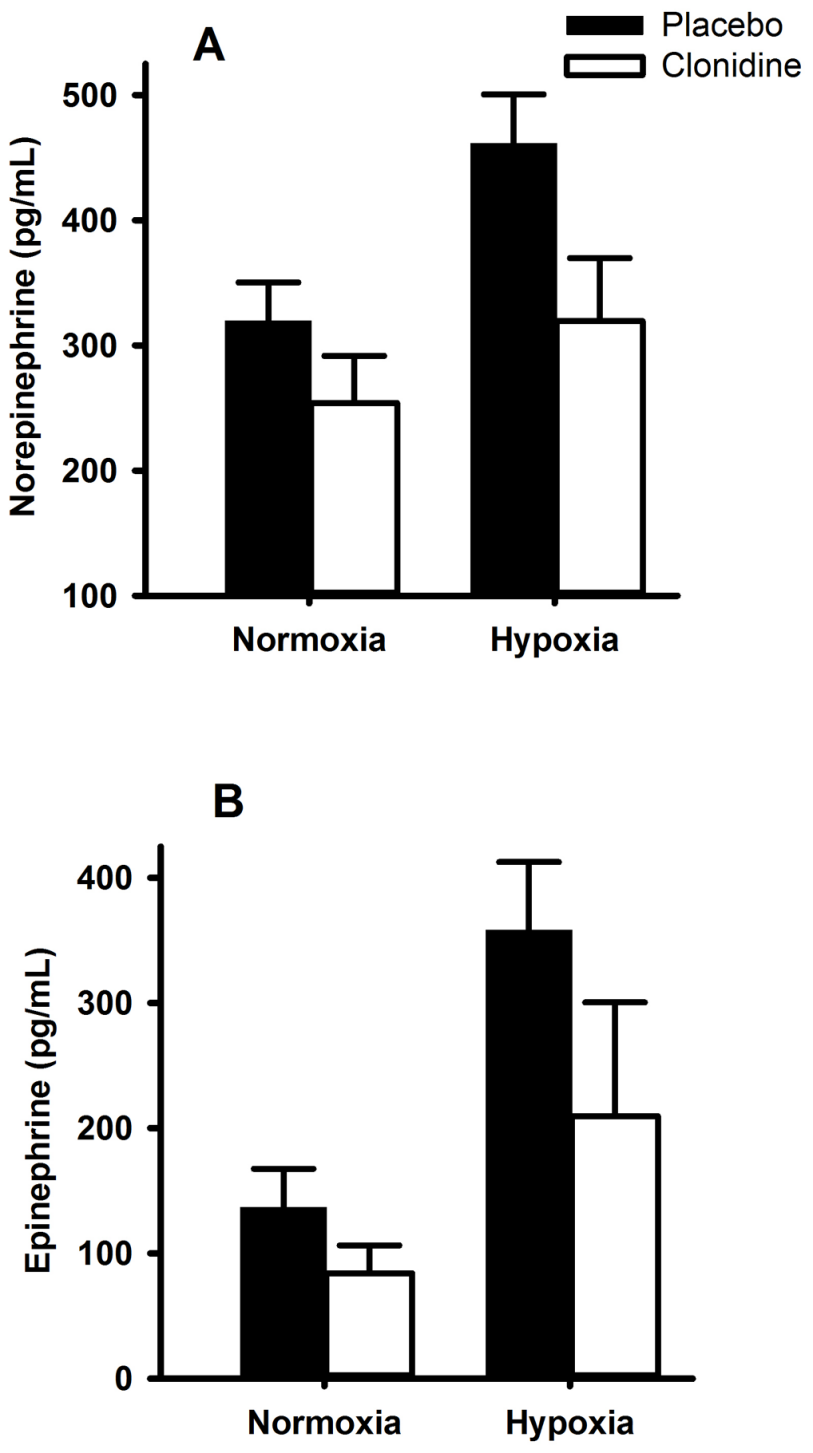
Circulating catecholamine response to hypoxia and clonidine. Plasma norepinephrine (A) and epinephrine (B) in response to normoxic (F_IO2_  = 0.21) and hypoxic gas (F_IO2_  = 0.11) breathing with and without concomitant sympathetic inhibition (48-h transdermal administration of the centrally acting α2-adrenergic receptor agonist clonidine). Data are mean ± SE. Hypoxic gas breathing increased norepinephrine (*P* = 0.001) and epinephrine (*P*<0.001). Clonidine attenuated the rise in norepinephrine (*P* = 0.014) and epinephrine (*P* = 0.061).

Clonidine decreased basal sympathetic activity, as indicated by decreases in circulating plasma norepinephrine (20%) and epinephrine (38%; Figure 1), and indirectly by decreases in heart rate (*P* = 0.001; Table 2) and diastolic blood pressure (*P* = 0.041; Table 2). This decrease in basal sympathetic activity was not accompanied by a change in plasma FGF21 (*P* = 0.434) or irisin (*P* = 0.418), suggesting that basal sympathetic activity does not contribute to the regulation of either variable.

**Table 2:** Hemodynamic responses to hypoxia with and without clonidine.

	Normoxia	Normoxia	Hypoxia	Hypoxia
	Placebo	Clonidine	Placebo	Clonidine
HR	57±2	49±3^*^	83±6^++^	71±4^**^^+^
SBP	123±3	113±3	126±5	120±5
DBP	75±3	66±2^*^	74±3	68±4

Data are mean ± SE. HR: Heart rate. SBP: Systolic blood pressure. DBP: Diastolic blood pressure. ^*^ *P*<0.05 compared to Normoxia – Placebo. ^**^ *P* = 0.061 compared to Hypoxia – Placebo. ^+^ *P*<0.001 compared to Normoxia – Clonidine. ^++^ *P* = 0.001 compared to Normoxia – Placebo.

**Figure 2:**
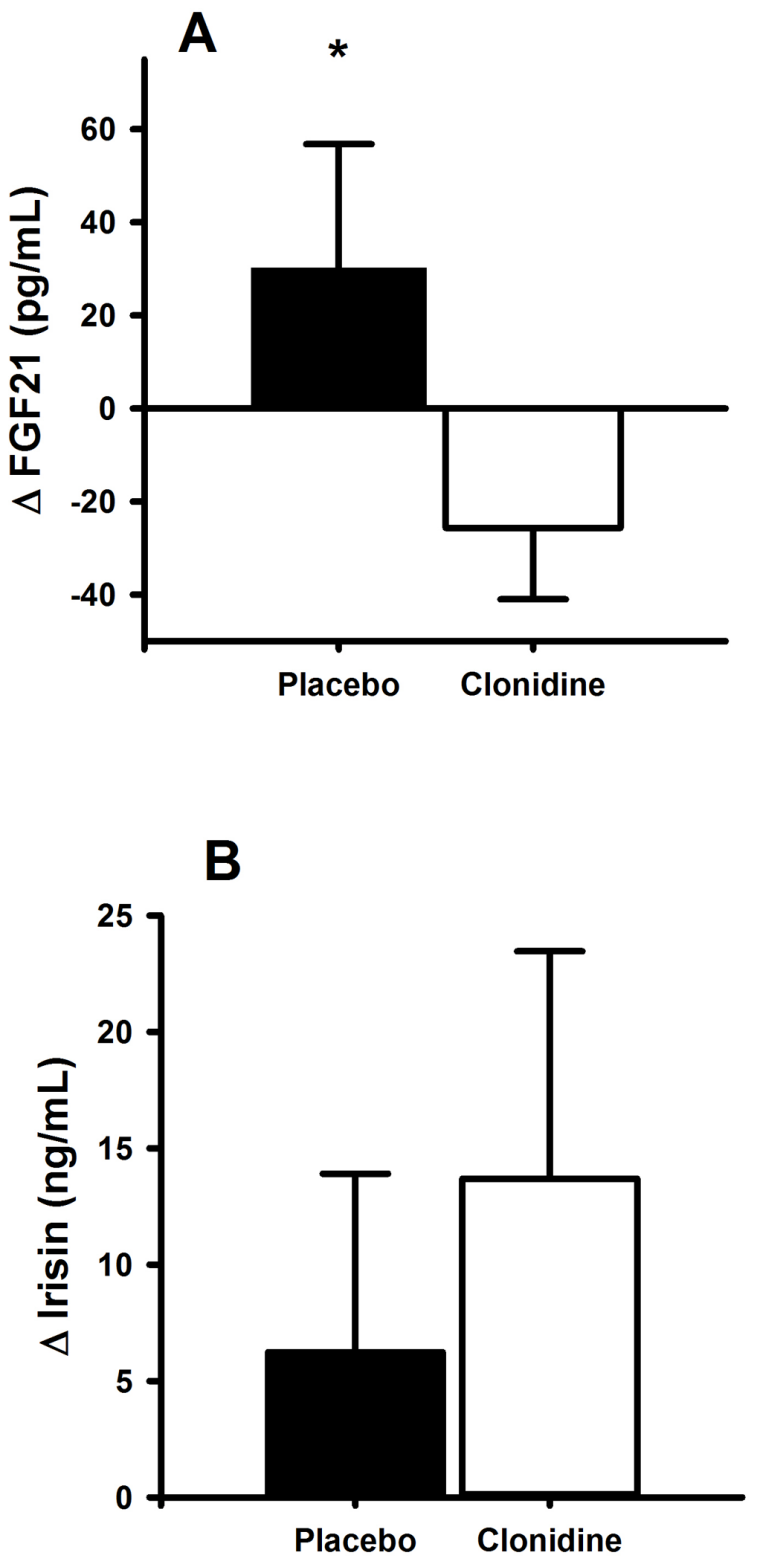
Sympathetic nervous system control of brown fat regulators. Change in plasma fibroblast growth factor 21 (FGF21) (A) and irisin (B) from normoxia (F_IO2_  = 0.21) to hypoxia (F_IO2_  = 0.11) with and without prior/concurrent sympathetic inhibition (48-hr transdermal administration of the centrally acting α2-adrenergic receptor agonist clonidine). Data are mean ± SE. * *P* = 0.035 compared to clonidine.

Hypoxia decreased resting oxyhemoglobin saturation from 96.7±0.4 to 62.0±2.1% (*P*<0.001); clonidine did not influence this response (*P* = 0.44). Hypoxia also dramatically increased sympathetic activity, as represented by 2-3-fold increases in circulating plasma norepinephrine and epinephrine (both *P*<0.001; Figure 1); and appreciable increases in heart rate and systolic blood pressure (Table 2). FGF21 was unaffected by basal sympathetic inhibition (338±113 *vs*. 295±80 pg/mL; *P* = 0.433), but was increased during hypoxia mediated sympathetic activation (368±135 pg/mL); this response was abrogated (Figure 2; *P* = 0.035) with clonidine (269±93 pg/mL), suggesting that plasma FGF21 responds to acute increased signaling from the sympathetic nervous system. Noteworthy, the hypoxia-mediated increase in plasma epinephrine was positively associated with the magnitude of increase in FGF21 (r = 0.90, *P* = 0.04). Irisin did not respond to the hypoxia-mediated increase in sympathetic activation; clonidine did not interact with this response (*P* = 0.438; Figure 2).

### Study 2 - Influence of short-term sprint interval training on skeletal muscle FNDC5 protein content and circulating FGF21 and irisin

**Figure 3:**
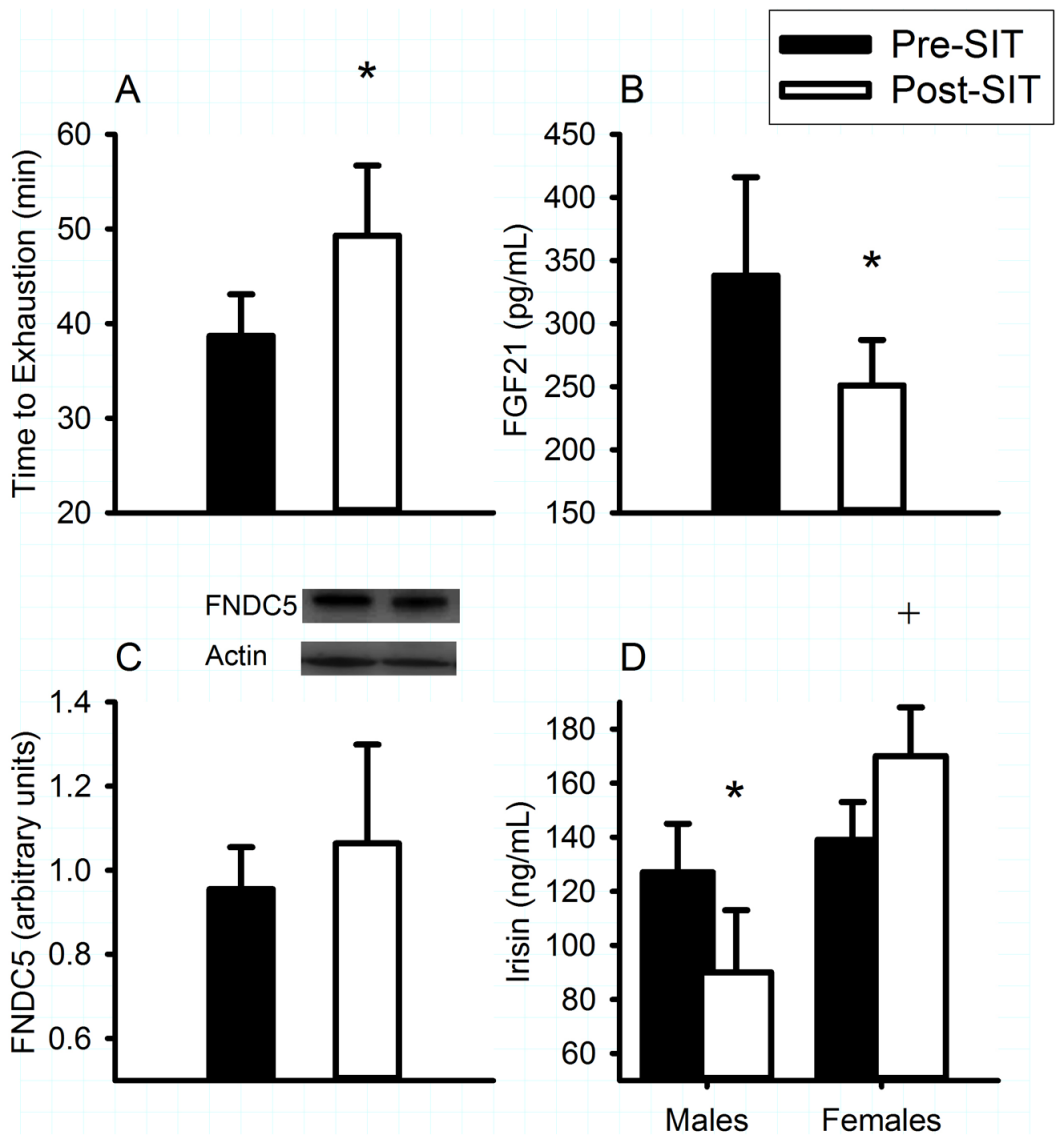
Effect of sprint interval training on exercise performance and brown fat regulators. Time to exhaustion (A), plasma fibroblast growth factor 21 (FGF21) (B), fibronectin type III domain containing 5 (FNDC5) protein expression (C), and plasma irisin (D) prior to (Pre-SIT) and following (Post-SIT) three weeks of sprint interval training (SIT). Data are mean ± SE * *P*<0.05 compared to Pre-SIT. + *P* = 0.001 compared to males Post-SIT.

Sprint interval training increased time to exhaustion (from 38.7±4.4 to 49.3±7.4 minutes, *P* = 0.048; Figure 3A) providing evidence that the exercise regimen was a physiologically significant intervention. Contrary to our hypothesis, sprint training decreased circulating FGF21 (*P* = 0.046; Figure 3B). Skeletal muscle FNDC5 protein content and plasma irisin were unaffected (both *P*>0.787) by sprint interval training.

In contrast to Study 1, Study 2 consisted of both male and female research participants, thus we were able to preliminary exploration of potential sex differences. With respect to the influence of sprint interval training on time to exhaustion, there was neither a main effect of sex (*P* = 0.793) nor an interaction between sex and training (*P* = 0.587), suggesting that males and females benefitted similarly from the exercise regimen. Consistent with this observation, the decrease in plasma FGF21 with sprint interval training was not sex-dependent (main effect of sex: *P* = 0.386; sex-training interaction: *P* = 0.894). Further, the lack of influence of sprint training on skeletal muscle FNDC5 protein was consistent between sexes (main effect of sex: *P* = 0.157; sex-training interaction: *P* = 0.629). Unexpectedly, there was a sex-training interaction for plasma irisin concentration (*P* = 0.012). Sprint interval training increased plasma irisin concentration in females and decreased it in males (Figure 3D). To further illustrate this sexual dimorphism, while there were no sex differences in plasma irisin at baseline, following sprint training circulating irisin was greater in females compared with males (*P* = 0.001).

To advance the exploration of the associations between these regulators of white adipose browning and indices of insulin resistance, the relations between FGF21, irisin and FNDC5 and fasting glucose, insulin, HOMA-IR and PEDF were examined. None of these relations were significant (all *P*>0.223) with the exception of an inverse relation between irisin and PEDF (r = −0.46, *P* = 0.047).

## Discussion

FGF21 and irisin/FNDC5 have been mechanistically linked to the conversion of white adipose to thermogenic beige adipose tissue [5], [10], however the physiological control of these regulators is both important to clarify and, at the moment, poorly understood. The novel findings of this manuscript are: 1) basal sympathetic activity does not influence circulating FGF21 or irisin; 2) FGF21 is increased in response to acute sympathetic activation; 3) sprint interval training decreases FGF21, does not affect skeletal muscle FNDC5, and results in a sexual dimorphic response in systemic irisin (reduced in males and increased in females).

The sympathetic nervous system is a well-known activator of beige (brown) adipose in adult humans. Combined use of positron emission tomography and computed tomography (PET/CT) scanning during administration of fluorodeoxyglucose (^18^F) is currently the gold standard non-invasive method of quantifying beige fat activation [24]; β-adrenergic receptor blockade decreases the metabolic activity of beige fat, thus rendering its detection extremely difficult [25]. It seems plausible that the sympathetic nervous system may also contribute to the regulation of FGF21 and/or irisin/FNDC5 as part of the coordinated control of the “browning” of adipocytes. Data from cell and animal studies support this notion. Administration of a non-selective β-adrenergic receptor agonist increases FGF21 mRNA and secretion [4], [5] in rodents, and β_3_-adrenergic receptor stimulation increases FGF21 gene expression in the white and brown adipose tissue of mice [5]. In humans, 60 minutes following acute exercise, and presumably acute sympathetic activation, circulating FGF21 is increased [16]. Further, acute exercise is a powerful stimulator of skeletal muscle PGC1-α [26], [27], mediated in part by sympathetic activation [28], and downstream targets of PGC1-α include FNDC5 and subsequently irisin [10]. Accordingly, we directly assessed the influence of tonic sympathetic activity and the responses to acute sympathetic activation of circulating FGF21 and irisin in adult men. Decreasing basal sympathetic activity did not influence FGF21 or irisin, however, consistent with cell and animal studies, FGF21 increased in response to acute sympathetic activation. Noteworthy, the magnitude of increase in circulating FGF21 was positively related to the magnitude of increase in circulating epinephrine. Peroxisome proliferator-activated receptor alpha (PPARα) in the liver and PPARγ in white adipose tissue are both activators of FGF21 [29]–[32], and, in turn, both will respond to the increase in free fatty acids resulting from sympathetically mediated lipolysis [33]. It is plausible that the increase in FGF21 we report was due to sympathetic activation and the subsequent lipolysis. From a teleological perspective, weight (fat) gain is associated with increased sympathetic activity [34]–[39], usually accompanied by increased β-adrenergic receptor mediated energy expenditure [40] to defend body composition. It seems feasible, at least initially (that is, before obesity associated β-adrenergic receptor dysfunction [41]–[43]), this increased sympathetic drive may also be targeting activation *and* formation of thermogenic adipose tissue.

We surmised that sympathetic activation may also contribute, at least in part, to the previously reported responses of FGF21 and irisin/FNDC5 to exercise training. This was based on previous studies reporting increases in all of these potential regulators following either acute or short-term exercise training in animals [10], [16] and humans [10], [16]–[18]. Sprint-interval training is a low-volume, high-intensity alternative to traditional, endurance exercise training. It has been shown repeatedly to evoke favorable and significant physiological adaptations that have positive implications for both health (cardiovascular and metabolic) and athletic performance [20], [44]–[47]. While sympathetic activation following sprint-interval training was not assessed in the current study, previous studies in humans confirm that sprint exercise activates the sympathetic nervous system [48]. In the current study, sprint interval training decreased circulating FGF21 and did not affect skeletal muscle FNDC5 protein content. However, circulating irisin was decreased in males and increased in females. To our knowledge, this is the first investigation to report on the influence of exercise training on FNDC5 protein content in human skeletal muscle. Ten weeks of endurance exercise increased FNDC5 mRNA [10] in obese men while FNDC5 mRNA did not change following 21 weeks of endurance exercise in normal weight men [49]. In animal models, exercise training increased FNDC5 mRNA in mice [10], but decreased FNDC5 mRNA and protein content in pigs [50]. In C2C12 myotubes, administration of AICAR, an exercise mimetic, decreased FNDC5 mRNA [51]. These discrepancies may be due to species differences, variations in exercise intensity and/or duration of exercise training, timing of tissue collection with respect to the most recent exercise bout, and/or kinetic differences between the mRNA and protein responses to exercise. A definitive explanation is beyond the scope of the current study.

Contrary to our hypothesis, circulating FGF21 was decreased following sprint interval training. In adult humans, previous studies have demonstrated increased circulating FGF21 following single bouts of exercise [16], greater magnitudes of increase in FGF21 following higher intensity exercise (80 *vs*. 50% VO_2max_) [16], and increased FGF21 after short-term (2-weeks) of incremental treadmill exercise [18]. In the acute exercise studies, blood was sampled one hour following exercise completion, and in the training study within 24-hours of the final exercise session. Recently, the half-life of FGF21 has been established as less than two hours in humans [52]. In the present study, blood was sampled 48-hours after the final exercise bout; thus discrepancies between the present and previous studies may be attributable to FGF21's short half-life and/or the duration for which its secretion was increased. An additional consideration pertaining to the FGF21 response to exercise training is the response of its co-factor, β-Klotho. β-Klotho is a member of the Klotho family of transmembrane proteins, is present in FGF21 target tissues, and is thought to be required for FGF21 mediated metabolic effects [53], [54]. Bidirectional FGF21 and β-Klotho responses to exercise and caloric restriction (that is, decreased FGF21 and increased β–Klotho) have been reported [55]; these responses were thought to mediate protection from obesity and obesity-induced nonalcoholic fatty liver disease in rats. Clearly the responses of, and interactions with, FGF21 to exercise training, including sprint interval training, are complex, and single time point studies may be inadequate to fully describe this physiology.

Another novel finding of the present study was the sexual dimorphic response of circulating irisin to sprint interval training. Recent studies have reported no change in circulating irisin following aerobic and strength training programs [49], [56], [57] and there were no differences in the responses to training between males and females [56]. Explanations for this current sex difference are potentially related to differences in the transcription/translation of FNDC5 and/or the regulation of the cleavage, secretion, and/or clearance of irisin. Potential contributors include differences in body composition, variability in other adaptations to sprint interval training (such as exercise tolerance), and the influence of circulating sex hormones. With respect to body composition, large population studies that have included adults spanning a wide range of body composition (anorexia through to obesity) have reported positive relations between circulating irisin and fat free mass [17], [58]. In the present study, fat free mass was lower in females compared with males but there was no relation between fat free mass (or any other index of body composition) and circulating irisin (data not shown). Relative to these other studies, our research participants comprised a smaller and relatively homogenous population; this may explain the non-significant associations. With respect to the influence of sprint interval training on exercise tolerance, there was neither a main effect of sex nor an interaction between sex and training, suggesting that males and females benefitted similarly, at least from an athletic performance perspective, from the exercise regimen. Finally, we did not attempt to time standardize data collection relative to menstrual phase. This decision was based on the hypothesis that the accumulative influence of sprint interval training would be greater than the influence of circulating sex hormones. Further, a sex hormone mediated explanation for the sexual dimorphic irisin response to sprint interval training seems unlikely as presumably the sex hormonal profile in the female participants would have been highly variable on account of the absence of menstrual phase standardized data collection. Thus, that a sexual dimorphic response was identified against the background of highly variable circulating sex hormone concentrations speaks to the strength of the dimorphic response.

Circulating irisin and FGF21 have been linked statistically with indices of insulin resistance [6]–[10], [59]. No significant relationships were discovered at baseline or post-sprint interval training among primary outcome variables and glucose, insulin, or HOMA-IR. Again, this may be reflective of the relatively homogenous study population coupled with the good health status of the research participants. We report for the first time on the inverse association between irisin and PEDF. This inverse association is consistent with the current understanding of the respective roles of PEDF and irisin on insulin sensitivity [20], [60]. Whether the relation between these two variables is independent of co-variables remains to be seen.

There are a few additional issues pertaining to these studies that warrant brief discussion. The first pertains to our choice of hypoxia as a method of evoking a sympathetic response. Alternatives to hypoxia include cold exposure, pharmacological manipulation (adrenergic agonists), and/or exercise. Cold exposure is already known to increase the thermogenic behavior of brown adipose in humans [3], [13], [14], [61], thus we chose a sympathetic activator not previously associated with brown adipose behavior. To inhibit basal sympathetic activation we chose clonidine. Use of hypoxia to activate the sympathetic nervous system avoided the possibility of potentially unfavorable pharmacological interactions. While exercise is a powerful sympathetic stimulator it evokes many other physiological responses making definitive interpretation as to control of irisin and FGF21 problematic. Of course, acute hypoxia is not without its own side effects, including inflammation and oxidative stress, however the fact that sympathetic inhibition with clonidine diminished the influence of hypoxia on the primary outcomes suggests that hypoxia per se, is not a significant controller. Next, the research participants in the current studies comprised young, healthy, non-obese adults. Obesity is known to modify/inhibit skeletal muscle and adipose function, thus it is possible that obese adults may not have responded in the same way (magnitude and/or direction) to the stimuli described herein. However, if one considers the law of initial baseline, then one might speculate that adults with low basal FGF21 and irisin/FNDC5 may have greater opportunity for improvement (that is, they will not be limited by a ceiling effect). Clearly a prospective empirical study would provide the most insight into this issue. Another potential limitation is the absence of a sedentary/time control condition in Study 2, the sprint interval training study. It is possible that the observed responses with respect to our primary outcome variables could be attributed to external factors. We think this is unlikely as there is no reason to suspect any of the variables would have changed; however, the limitation is acknowledged. Finally, the decision to investigate sex differences in study 2 was made *a posteriori.* Accordingly, the design was potentially underpowered to discern differences between males and females in FGF21 and FNDC5. However, this speaks to the strength of the discovery of the sexual dimorphic response of irisin to sprint training.

In summary, the conversion of white adipose tissue to the highly thermogenic beige adipose tissue in adult humans has been proposed as a potential treatment for global obesity [1]–[3]. Three regulators of this conversion have recently been described but information regarding their control is limited, and somewhat contradictory. Our data suggest while basal sympathetic activity does not influence circulating FGF21 or irisin, FGF21 is increased in response to acute sympathetic activation. Also, although sprint interval training does not affect skeletal muscle FNDC5 protein content, it does decrease circulating FGF21 and has opposing effects on circulating irisin in males and females.
